# Liver Cancer-Derived Hepatitis C Virus Core Proteins Shift TGF-Beta Responses from Tumor Suppression to Epithelial-Mesenchymal Transition

**DOI:** 10.1371/journal.pone.0004355

**Published:** 2009-02-03

**Authors:** Serena Battaglia, Nassima Benzoubir, Soizic Nobilet, Pierre Charneau, Didier Samuel, Anna Linda Zignego, Azeddine Atfi, Christian Bréchot, Marie-Françoise Bourgeade

**Affiliations:** 1 Inserm, Unité 785, Villejuif, France; 2 Univ Paris-Sud, UMR-S 785, Villejuif, France; 3 Institut Pasteur, Groupe de Vectorologie, Paris, France; 4 AP-HP Hôpital Paul Brousse, Centre Hépato-Biliaire, Villejuif, France; 5 Department of Internal Medicine, University of Florence, Florence, Italia; 6 Inserm Unité 673, Paris, France; Emory University, United States of America

## Abstract

**Background:**

Chronic hepatitis C virus (HCV) infection and associated liver cirrhosis represent a major risk factor for hepatocellular carcinoma (HCC) development. TGF-β is an important driver of liver fibrogenesis and cancer; however, its actual impact in human cancer progression is still poorly known. The aim of this study was to investigate the role of HCC-derived HCV core natural variants on cancer progression through their impact on TGF-β signaling.

**Principal Findings:**

We provide evidence that HCC-derived core protein expression in primary human or mouse hepatocyte alleviates TGF-β responses in terms or growth inhibition or apoptosis. Instead, in these hepatocytes TGF-β was still able to induce an epithelial to mesenchymal transition (EMT), a process that contributes to the promotion of cell invasion and metastasis. Moreover, we demonstrate that different thresholds of Smad3 activation dictate the TGF-β responses in hepatic cells and that HCV core protein, by decreasing Smad3 activation, may switch TGF-β growth inhibitory effects to tumor promoting responses.

**Conclusion/Significance:**

Our data illustrate the capacity of hepatocytes to develop EMT and plasticity under TGF-β, emphasize the role of HCV core protein in the dynamic of these effects and provide evidence for a paradigm whereby a viral protein implicated in oncogenesis is capable to shift TGF-β responses from cytostatic effects to EMT development.

## Introduction

Epithelial to mesenchymal transition (EMT) is defined as a process in which epithelial cells lose their phenotypic characteristic and acquire mesenchymal cell's features. While EMT is involved in the context of embryonic development it also plays a role in the genesis of fibroblasts during organ fibrosis in adult tissues and might contribute to the metastatic carcinoma development [1]. Indeed, EMT is being increasingly recognized as a crucial step that promotes cell migration, tumoral invasiveness and metastasis [2] and has also been implicated recently in cancer stem cell emergence [3]. In the liver, hepatic stellate cells (HCS) are considered as the major fibrotic precursor cells that transdifferentiate to fibrogenic, extracellular matrix producing myofibroblasts in inflammatory liver tissue upon TGF-β signaling, whereas hepatocytes undergo apoptosis upon signaling by this cytokine. However, identification of different fibrogenic populations apart of resident stellate cells [4] as well as convergent results of recent studies have challenged the paradigm of HSC as the essential source of liver myofibroblasts and inferred a prominent role for hepatocytes in liver fibrogenesis. Indeed, it has been reported recently that rat or mouse hepatocytes respond both *in vitro* and *in vivo* to TGF-β not only in terms of cell growth inhibition and apoptosis, but also in terms of induction of EMT [5]–[7]. Accordingly, it has been shown that TGF-β and laminin 5 transform non invasive hepatocellular carcinoma cells into invasive cells through induction of a complete EMT [8]. However, although the molecular mechanisms underlying EMT development have been studied extensively, little evidence is available concerning its physiological functions and relevance in human pathologies.

One of the mechanisms whereby cells undergo neoplastic transformation and escape from normal growth control involves an altered response to the cytostatic effects of TGF-β [9], [10]. Furthermore, during the later stages of tumorigenesis, TGF-β can stimulate invasion mainly through induction of EMT. It is now generally accepted that TGF-β has a dual role in oncogenesis and can act as a tumor suppressor or tumor promoter factor depending on cellular context [11], [12], but the mechanisms involved in the switch of TGF-β responses toward malignancy are not fully understood. *In vivo*, it has been shown that loss of TGF-β signaling significantly decreased tumor latency and increased the rate of metastasis in several mouse models [13].

TGF-β initiates responses by contacting two types of trans-membrane serine/threonine kinases called receptors type I and type II, promoting activation of the type I by the type II kinase. The activated type I receptor then propagates the signal to the nucleus by phosphorylating Smad2 and Smad3. Once phosphorylated, Smad2 and Smad3 associate with the shared partner Smad4 and the complexes accumulate in the nucleus where they regulate the expression of TGF-β target genes through cooperative interactions with transcriptional partners [14], [15], [16]. Disruption of TGF-β signaling, either via mutational inactivation of components of the signaling pathway, or by modulation of their expression or function, is now known to play an important role in tumor progression. Despite all these evidences, the clinical implication of TGF-β in metastasis progression remains unclear.

Chronic hepatitis C virus (HCV) infection and associated liver cirrhosis represent a major risk factor for hepatocellular carcinoma (HCC) development, and despite epidemiologic evidence connecting HCV infection to HCC, the clinical impact of this virus on hepatocarcinogenesis is still unclear [17]. Because HCV RNA shows high genetic variability, chronic HCV infection results in a complex population of different but closely related viral variants commonly referred as quasispecies [18], [19]. The non-random distribution of HCV quasispecies has been observed between tumoral and non-tumoral liver suggesting the possibility of a selection of quasispecies with modified functional properties that could contribute to fibrosis development as well as tumorigenesis process [20].

The structural component of HCV, HCV core protein has attracted particular attention after its characterization and various reports have suggested its potential role in HCV pathogenesis. Indeed, besides its role in viral RNA packaging, HCV core protein has been reported to interact with several cellular proteins such as TNFR [21], PKR [22], Stat3 pRB or p53 [23] leading to modulation of transcription of genes dependent on these cascades and consequently to modulation of a number of cellular regulatory functions. In fact, numerous data have suggested a possible involvement of HCV core protein in the modulation of cell proliferation and apoptosis although some results have been controversial given that core protein has been reported to exhibit pro or antiapoptotic effects depending on the experimental system used [24], [25]. Moreover these studies were mainly performed using apoptotic agents from the TNF family and not with TGF-β. This discrepancy could also be due to genetic heterogeneity of different HCV genotypes.

We and others have previously demonstrated an interaction between Smad3 and the HCV core protein [26], [27]. Interestingly, we also observed that different natural core variants isolated from tumor or non tumor nodules could differently bind Smad3, and consequently inhibit TGF-β induced Smad3 transcriptional activity suggesting that the HCV core protein may modulate TGF-β signaling and its downstream biological responses [27]. We hypothetised that the molecular heterogeneity of HCV observed in infected patients could be involved in the clinical course of cancer development.

Overexpression of TGF-β and concomitant decrease in hepatocyte growth inhibition is frequently observed in HCC supporting the notion that TGF-β could play a tumor promoting role in liver cancer [28]. However, the functional implication of TGF-β in liver tumorigenesis as well as the implication of EMT in HCC development are not yet elucidated. Likewise, effects of oncogenic viral hepatitis B or C proteins on EMT development have not been studied in the course of hepatocarcinoma process. Demonstrating interplay between HCV infection and TGF-β mediated EMT may provide a new model to gain insights in the mechanisms of liver carcinogenesis.

In this study, we made use of natural HCV core variants isolated from HCV-related HCC tissues to analyze their impact on the dual function of TGF-β in a pathophysiogically-relevant condition. Thus, we investigated the effects of core protein variants isolated from both tumor or non tumor cirrhotic areas in primary human hepatocytes; indeed, cirrhosis is a well-known preneoplastic condition, associated in at least 90% of cases of HCC. Using these variants we provide evidence for a paradigm in which a viral protein is capable to shift TGF-β responses from cytostatic effects to EMT development.

## Materials and Methods

### Materials

Recombinant TGF-β1 and recombinant TRAIL/Apo2L were purchased from Abcys, the chemical inhibitor of TGF-β signaling SB-431542 that acts by specifically interfering with the type I receptor [29] was from Calbiochem, the fluorescent dye DiOC_6_ (3,3′dihexylocarbocyanine iodide) was from Molecular Probes.

### Vectors

Full length HCV core sequences were amplified from HCV-RNA extracted from tumor (T) or cirrhotic (NT) nodules of a patient (patient B) infected with HCV 1b genotype as previously described [22]. PCR products were directly sequenced and inserted into the pcDNA3.1 vector. The sequence of these two variants has been previously described [27]. The T sequence differs from the NT one by 2 changes in aa 118 (N→D) and aa 189 (A→V).

(CAGA)_9_-Luc was kindly provided by Dr J.M. Gauthier. The expression vectors for HA-TβRI.act, and Flag-TβRImL45.act were a gift from Dr. Y.E. Zhang [**30**]. The pRetroSuper-puro plasmid containing short hairpins RNA antisense against Smad3 was kindly provided by Dr J. Massagué [**31**]. A pRetroSuper-puro plasmid containing scramble short hairpins RNA was used as control. pIRES-GFP was obtained from Stratagene, pCMV-Renilla-luc was from Promega. Myc-Smad3 expression vector was previously described [32].

### Transgenic mice

To obtain transgenic mice, the HCV core cDNAs isolated from tumor (T) or cirrhotic nodules (NT) were cloned downstream of hepatitis B virus regulatory elements and introduced into C57BL/6 embryos (Institut Clinique de la Souris, Strasbourg, France). Transgenic mice were identified by subjecting 1 µg of tail DNA to amplification by PCR.

### Cell culture

The human hepatoma cell line Huh7 [33] was maintained in Dulbecco Modified medium containing 10% fetal calf serum (FCS). Cells were transfected with the different vectors using the LipofectAMINE method (Invitrogen) and stable transfectants were selected by incubating the cells with the antibiotic corresponding to the selection gene.

### Isolation and culture of primary hepatocytes

Primary mouse hepatocytes were isolated by liver perfusion with a collagenase blend as previously described [34]. After isolation, hepatocytes were resuspended in Williams medium supplemented with 10% fetal calf serum, 100 µg/ml streptomycin, 100 U/ml penicillin, 250 ng/ml fungizone (“plating medium”) and plated at the density of 3×10^4^ cells/cm^2^. After 4 hours, serum-containing medium was removed and cells were cultured in Williams medium supplemented with 1 mg/ml bovine serum albumin, 100 µg/ml streptomycin, 100 U/ml penicillin, 250 ng/ml fungizone, and treated with TGF-β 2 ng/ml or SB431542 1 µM.

Primary human hepatocytes were isolated from the healthy liver tissue of surgical liver biopsy specimens collected after informed consent obtained from patient undergoing therapeutic partial hepatectomy for liver metastasis and benign hepatic tumor. Collagenase (Sigma Aldrich) perfusion (500 µg/ml, 2.4 mg/ml CaCl_2_ in HEPES buffer, pH 7.4) was preceded by extensive washing of the liver tissue with HEPES/EDTA buffer (pH 7.4) using a catheter inserted into the vessels on the cut surface of the resected fragment. Cells were then washed twice and hepatocytes were separated from nonparenchymatous cells by Percoll fractionation (30% isotonic Percoll solution, centrifuged at 450 g for 4 min) and immediately infected at 37°C for 2 h with lentiviral vectors, washed and plated in Williams medium supplemented as described elsewhere [35]. Twelve hours later, they were treated or not with TGF-β or SB431542 for various periods of time.

### Lentiviral vectors

TRIP-ΔU3-CMV-T, TRIP-ΔU3-CMV-NT and TRIP-ΔU3-CMV-Cinv vectors were obtained by substituting GFP in TRIP-ΔU3-CMV-GFP with cDNA coding for HCV core sequences. An inverted core sequence TRIP-ΔU3-CMV-Cinv was used as a control.

Vector particles were produced by the transient calcium phosphate cotransfection of 293T cells as a previously described [36]. Vector concentrations were normalized according to the p24 (HIV-1 capsid protein) content of supernatants.

### Western blotting

Cells were washed twice with PBS and lysed in RIPA buffer containing 0.5% SDS and Benzon nuclease. Proteins were quantified with the Bio-Rad protein assay (Bio-Rad, France) and 30 µg of extracts were separated on SDS polyacrylamide gel, transferred on nitrocellulose membrane and blotted using different primary antibodies directed against HCV core protein, E-cadherin, Fibronectin (Santa Cruz Biotechnology), Vimentin (Chemicon), phospho-Smad3 (Cell signaling), Smad3 (Abcam), Flag, Myc and HA tags (Sigma). Membranes were revealed using a chemioluminescence detection kit (ECL Plus, GE Healthcare).

### Cell staining

Primary mouse hepatocytes were cultured for 48 h with or without TGF-β (2 ng/ml) and routine stain hematoxylin-eosin was performed after fixation of cells with EtOH 70% at 4°C for 15 min.

### Immunofluorescence staining

Cells were washed with PBS and fixed with a 4% PFA solution at 4°C for 20 min followed by methanol permeabilization for 5 min at −20°C. Cells were then incubated with a primary mouse anti-vimentin, rabbit anti-αSMA, or rabbit anti-E-cadherin antibody and then with an Alexa Fluor 488 conjugated anti-mouse antibody and an Alexa Fluor 594 conjugated goat anti-rabbit antibody (Molecular Probes). They were then stained with Hoechst and examined by fluorescence microscopy.

### Cell proliferation and apoptosis assays

Cell proliferation was assessed by BrdU incorporation (Roche), cell viability and caspase 3 activity were estimated using a Celltiter-Glo luminescent cell viability assay or the CaspaseGlo 3/7 assay respectively (Promega) according to the manufacturer's instructions.

Mitochondrial transmembrane potential (ΔΨm) was evaluated by staining cells (10^6^) with the fluorescent dye DiOC_6_ at a final concentration of 40 nM for 15 min at 37°C. Cells were immediately dissociated by trypsin and their fluorescence estimated by analysis with a FACScan flow cytometer (Becton-Dickinson) using the FL1 channel [37].

### Cell sorting

Flow cytometric analysis and sorting were performed using a FacsDiva flow cytometer (Becton Dickinson Immunocytometry Systems). Forward Scatter (FSC) and side scatter (SSC) were collected through a filter. The GFP signal was collected in the FL1 channel. A light gate was drawn in the SSC versus FSC to exclude dead cells/debris. Cells in the gate were displayed in a biparameter histogram (FS versus FL1) and final gating settings determined to collect the labeled cells. GFP positive cells were sorted at 5000 cells/sec.

### Transcriptional analysis

Cells were cotransfected with vectors coding for the gene of interest together with the CAGA-luc reporter plasmid and the Renilla luciferase plasmid to normalize the results. They were incubated 24 h later in the absence or presence of TGF-β for another 18 h. Luciferase activity was measured with the Dual Luciferase reporter assay (Promega) system according to the manufacturer's instructions.

### Statistical analysis

The significance between the different conditions and their control was determined by paired Student's *t* test using GraphPad Prism software. A p-value≤0.05 was considered significant.

## Results

### HCV core variants alleviate TGF-β cytostatic responses and increase TGF-β -mediated EMT in mouse or human primary hepatocytes

**Figure 1:**
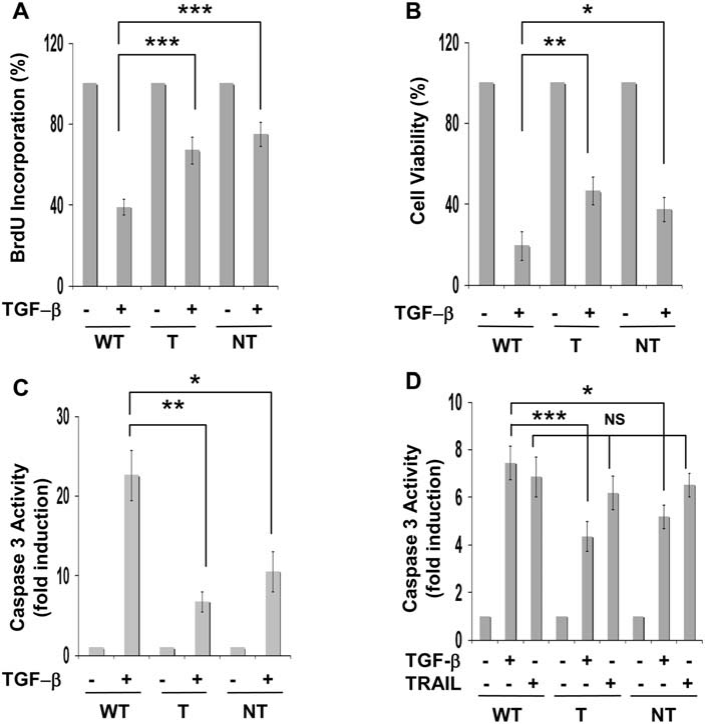
Expression of HCV core proteins in primary mouse hepatocytes reduce cell growth inhibition and apoptosis induced by TGF-β. (A,B,C) Mouse hepatocytes obtained from livers of transgenic mice expressing or not HCV core proteins isolated from tumor (T) or cirrhotic (NT) tissues were treated with TGF-β for 48 h before determination of cell proliferation, estimated by BrDU incorporation (A), cell viability (B) or caspase 3 activity (C). (D) Cells were treated with TRAIL (20 ng/ml) for 18 h before determination of caspase3 activity. Results represent the mean+/−SD of triplicates from a representative experiment. * p≤0.05, ** p≤0.005, *** p≤0.0005.

We have previously demonstrated that, when transiently expressed in hepatic cells, HCV core proteins isolated from tumor or cirrhotic nodules bind Smad3 differently and that this interaction inhibits Smad3-dependent transcriptional activity [27]. To ascertain the physiological relevance of this observation, we first investigated the impact of such binding on TGF-β biological responses in hepatocytes isolated from transgenic mice expressing these HCV tumor (T) or cirrhotic (NT) core variants under the control of the HBx promoter and which is mostly expressed in the liver. Hepatocytes were isolated from livers of 2 month old mice and treated or not with TGF-β for 48 hours. We observed that TGF-β was less potent to inhibit cell proliferation in hepatocytes isolated from transgenic mice expressing the HCV core proteins than in hepatocytes isolated from a control mouse (Fig. 1A). Accordingly, cell viability was less reduced by TGF-β in cells expressing the core proteins as compared to wild type cells (Fig. 1B). We also found that expression of the HCV core proteins inhibited TGF-β-mediated apoptosis as shown by caspase 3 activation, which represents a well-defined hallmark of apoptosis (Fig. 1C). Interestingly, T core expression decreased TGF-β -mediated apoptosis or inhibition of cell viability to a higher extent than the NT core showing a functional significance of the increased interaction of this core variant with Smad3 [27]. In order to verify that this HCV core-induced reduction of apoptosis observed after TGF-β treatment was specific, we used another inducer of apoptosis, TRAIL. Mouse hepatocytes expressing or not the HCV core proteins respond to TRAIL in a similar manner in terms of caspase 3 activation suggesting that the overall apoptosis process was not modified by core expression (Fig. 1D). This result is in agreement with a previous report indicating that HCV core leads to TRAIL-induced apoptosis through activation of the mitochondrial-signaling pathway [38].

**Figure 2:**
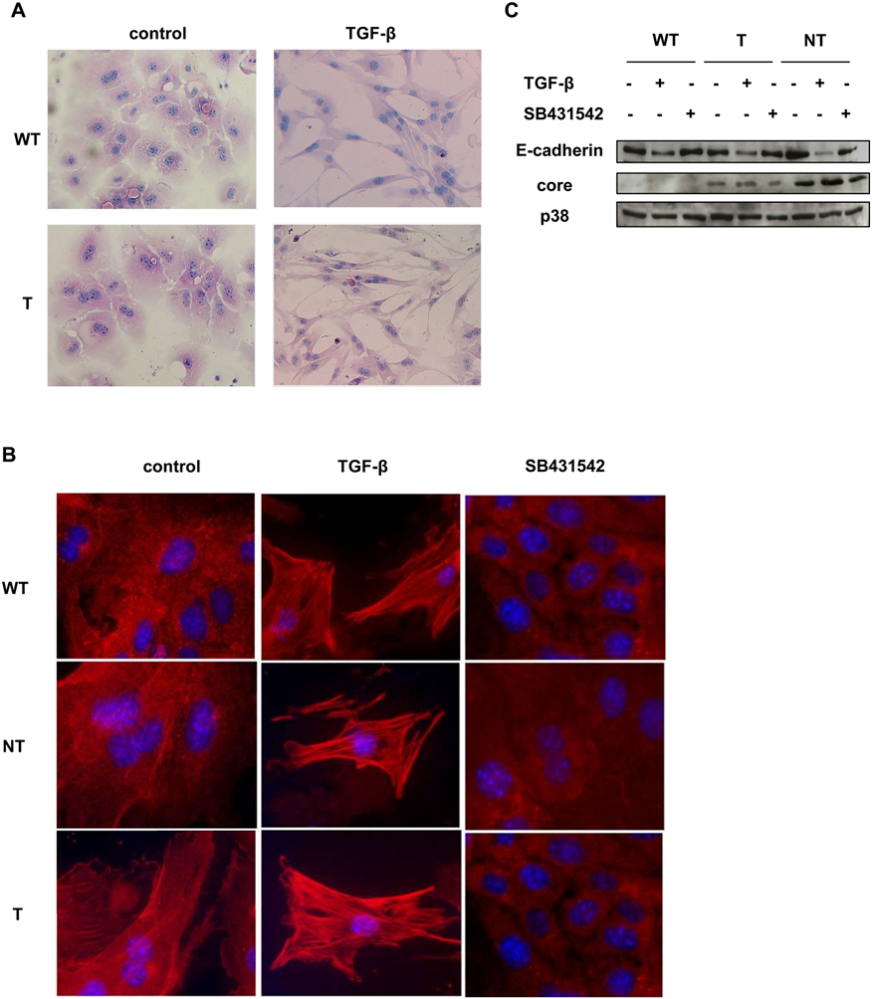
Expression of HCV core proteins in primary mouse hepatocytes increase EMT induced by TGF-β. (A) Morphologic changes of mouse hepatocytes expressing or not HCV T core protein observed after 48 h of culture with or without TGF-β (2 ng/ml). (B) Hepatocytes isolated from transgenic mice expressing HCV core proteins were treated with TGF-β (2 ng/ml) or SB431542 (1 µM) for 48 h and expression of αSMA was examined by immunofluorescence using a αSMA antibody. Data are representative of three independent experiments. (C) Hepatocytes isolated from transgenic mice expressing HCV core proteins were treated with TGF-β or SB431542 for 48 h and expression of E-cadherin was determined by Western blotting. Anti-p38 western blotting was used as control loading. Data are representative of three independent experiments.

Several lines of evidence support the notion that epithelial cancer cells lose their capacity to respond to TGF-β cytostatic effects but in some cases retain their ability to respond to other TGF-β -mediated functions such as EMT. The observation that HCV core proteins interfere with the ability of TGF-β to execute cell growth inhibition and cell killing prompted us to consider the possibility that these proteins might influence TGF-β mediated EMT. Since recent findings have demonstrated that TGF-β could induce an EMT in mature mouse hepatocytes *in vitro* [6], [7], we investigated whether HCV core proteins could modulate the ability of TGF-β to promote EMT in the same primary hepatocytes. Contrast microscopy observation revealed that after treatment for 30 h with TGF-β some hepatocytes acquired a fibroblast-like morphology suggestive of EMT and that this effect was more pronounced when these hepatocytes express the core protein showing that cell plasticity could be increased in mouse hepatocytes expressing HCV core T protein (Fig. 2A). This observation was reinforced by videomicroscopy observation (data not shown). To confirm that these observed phenotypic changes were reflective of an EMT, we performed immunofluorescence analyses on hepatocytes isolated from control or from transgenic mice. In line with previous findings, TGF-β treatment of control mouse hepatocytes was accompanied by a very strong increase in the polymerization of the mesenchymal marker alpha smooth muscle actin (αSMA) consistent with a phenotype of EMT (Fig. 2B). Interestingly, HCV core proteins and particularly the T one could increase the αSMA fibers in the absence of exogenously added TGF-β. To assess whether autocrine release of TGF-β could be involved in the formation of αSMA stress fibers in HCV core expressing cells, we used a specific TGFβR1 inhibitor, SB431542. When these expressing cells were treated with this inhibitor, αSMA fibers completely disappeared, suggesting that the effect of the core protein on EMT development is mediated by an endogenous production of TGF-β (Fig. 2B). In accordance, Western blots analyses also showed that E-cadherin expression, an epithelial marker known to be lost in mesenchymal cells, was greatly decreased by TGF-β and fully restored by addition of SB431542 (Fig. 2C).

**Figure 3:**
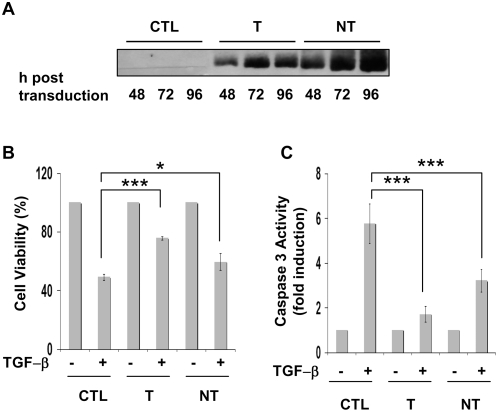
TGF-β cytostatic responses in primary human hepatocytes expressing HCV core proteins. Freshly isolated cells were infected with lentiviruses encoding the HCV core protein variants or an inverted core sequence as control (CTL) (A) Levels of core expression were estimated by Western blot analysis different time points after lentivirus transduction. (B, C) Determination of cell viability (B) or caspase 3 activity (C) was performed after 96 h of treatment with TGF-β (5 ng/ml). Results represent the mean+/−SD of triplicates from a representative experiment. * p≤0.05, *** p≤0.0005.

To obtain further evidence that HCV core proteins could modulate the magnitude of the negative growth regulatory effects of TGF-β we also performed experiments in human primary hepatocytes. Freshly isolated hepatocytes were infected with lentiviruses coding for the T or NT core variants or an inverted core sequence as control. Western blot analyses confirmed the expression of the core proteins (Fig. 3A). Cells were then treated or not with TGF-β for 96 h prior to analysis for cell viability or caspase 3 activation. Both TGF-β-mediated decrease in cell viability (Fig. 3B) and apoptotic responses (Fig. 3C) were alleviated by HCV core expression confirming the results obtained in mouse hepatocytes.

**Figure 4:**
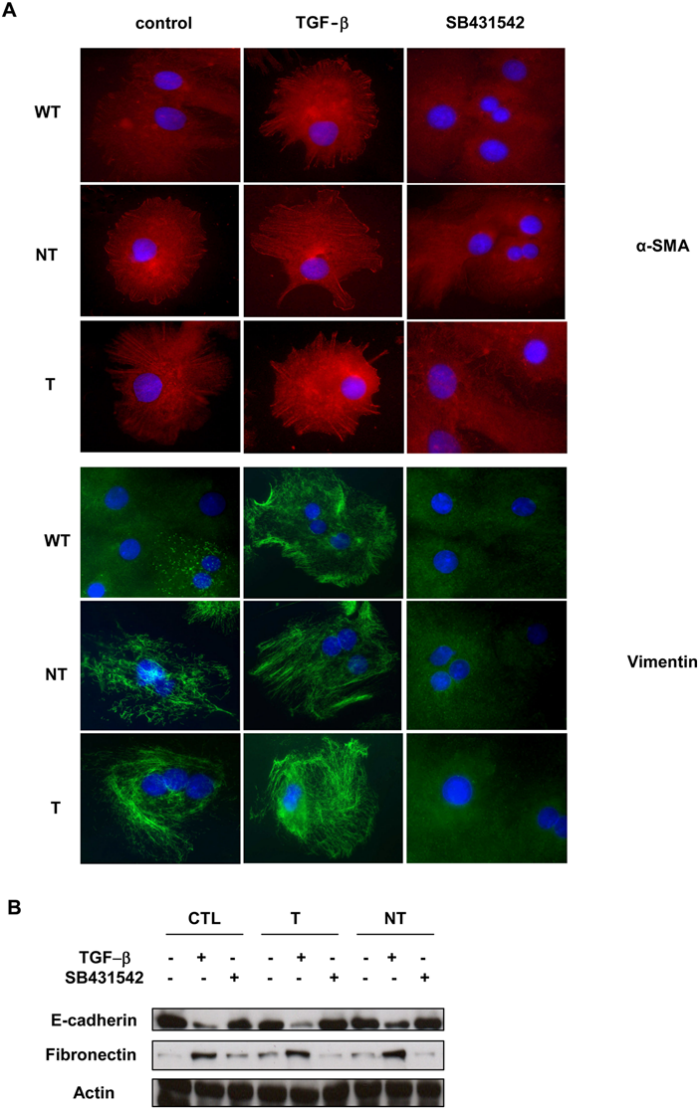
TGF-β increases EMT in primary human hepatocytes expressing HCV core proteins. (A) Expression of αSMA or Vimentin was estimated by immunofluorescence analysis after treatment with TGF-β (5 ng/ml) or SB431542 (1 µM). (B) Expression of Fibronectin or E-Cadherin was estimated by Western blot analysis in the same experimental conditions. Data are representative of three independent experiments.

Although TGF-β -mediated EMT has been described in primary mouse or rat hepatocytes as well as in cancerous human cells, no such study has been yet investigated in primary human hepatocytes in vitro. Interestingly, we observed that human hepatocytes could express stress fibers as spikes mainly located in membrane protrusions under TGF-β treatment (Fig. 4A). Expression of HCV core proteins increased this TGF-β effect. Here again expression of the HCV core proteins increased αSMA polymerization even in the absence of exogenously added TGF-β. This effect could involve endogenous TGF-β since it was completely abolished in the presence of the TGF receptor inhibitor. To corroborate this result, we studied the expression of another mesenchymal marker, vimentin. In accordance with the data obtained with αSMA, we observed that in control hepatocytes vimentin expression was markedly increased after TGF-β treatment and that this increase was greater when hepatocytes expressed the NT core protein and even greater when T core was expressed (Fig. 4A). Similarly, core proteins induced vimentin expression and polymerization in the absence of exogenously added TGF-β. This expression was completely reversed by the TGFbRI inhibitor suggesting again that endogenously produced TGF-β could be responsible for this effect.

Western blots analyses evidenced a lower expression of E-cadherin after TGF-β treatment which was totally recovered in the presence of the TbRI inhibitor. On the contrary, expression of the mesenchymal marker fibronectin was greatly increased by TGF-β (Fig. 4B).

Taken together these data strongly suggest that HCV core interfere with TGF-β responses in terms of cell growth inhibition and apoptosis in hepatocytes isolated from transgenic mice as well as human primary hepatocytes. Remarkably, TGF-β responses, in terms of EMT are increased by expression of T or NT core protein variants in both mouse and human hepatocytes. This might reflect both direct effects of core on TGF-β-induced EMT and reduction of TGF-β induced apoptosis by the core protein, allowing more cells to undergo EMT as compared to control cells.

### HCV core modulates TGF-β responses in Huh7 cells

**Figure 5:**
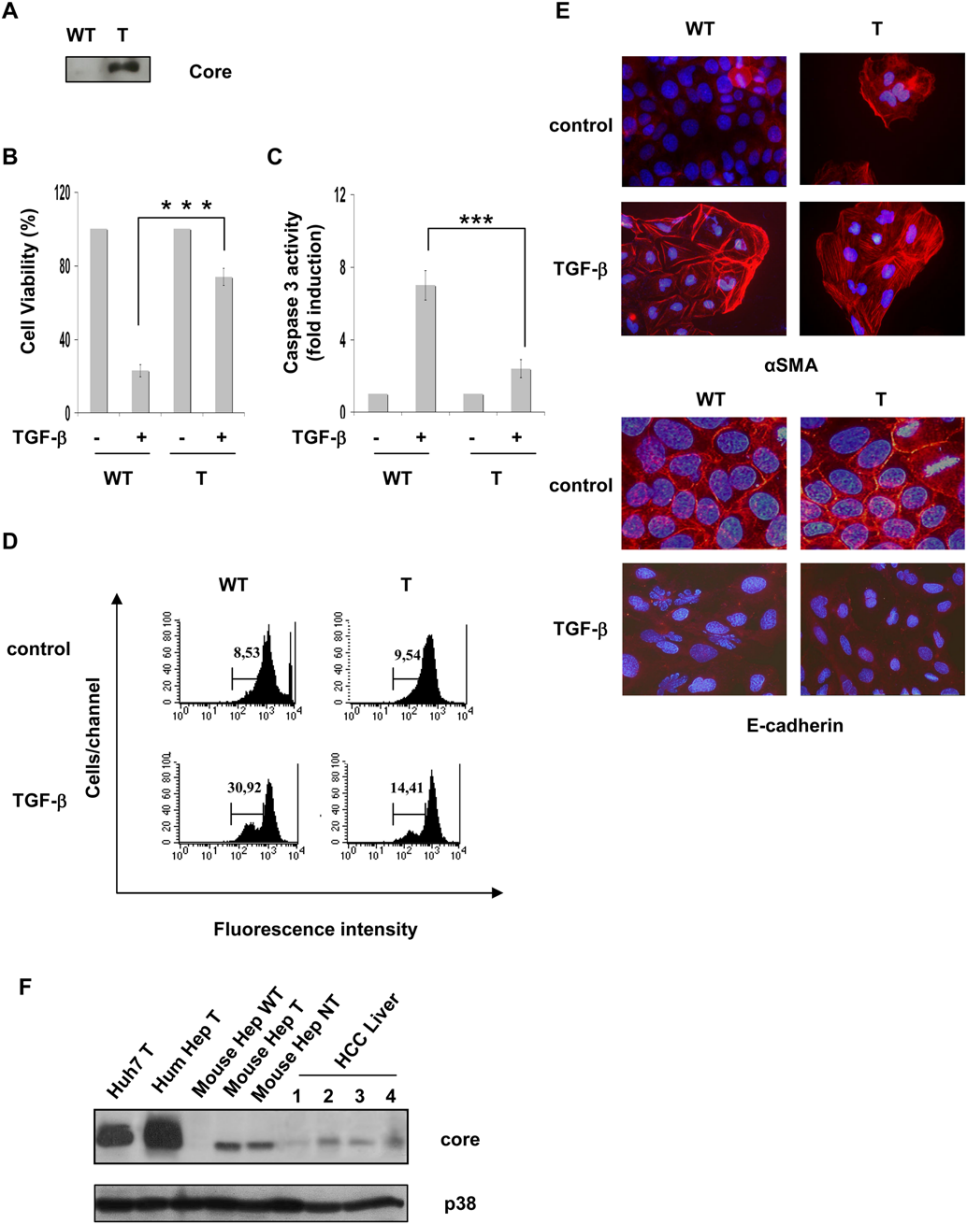
TGF-β responses in Huh7 cells stably expressing HCV core protein. (A) Expression of HCV core protein determined by Western blot analysis. (B, C) Cells were treated with TGF-β (5 ng/ml) for 48 h before determination of cell viability (B) or caspase3 activity (C). Results represent the mean+/−SD of triplicates from a representative experiment. *** p≤0.0005 (D) Mitochondrial membrane potential (ΔΨm) was estimated by FACS analysis in cells treated with TGF-β for 48 h. After staining with DiOC_6_(3), cells with low fluorescence intensity corresponding to low (ΔΨm) were gated and their number expressed as a percentage of the total population. A representative experiment is shown. (E) Cells were treated with TGF-β for 48 h and E-cadherin or αSMA expression was assessed by immunofluorescence analysis. Data are representative of three independent experiments. (F) Comparative expression of HCV core proteins. Extracts from cultured cells expressing the core protein (Huh7, human or mouse primary hepatocytes) or from livers of HCV-related HCC patients were analyzed by Western blot. Anti-p38 western blotting was used as control loading.

In order to dissect the molecular mechanisms activated by the HCV core protein, we established Huh7 cell lines stably expressing the T core protein (Fig. 5A). Core protein inhibited TGF-β-mediated Smad3 transcriptional activity measured by expression in these cells of a reporter plasmid, which contains CAGA elements previously shown to be transactivated by TGF-β through Smad proteins (not shown). Consistent with the results observed in primary hepatocytes, we found that HCV core protein was able to decrease the inhibitory effect of TGF-β on cell viability (Fig. 5B). Similarly, TGF-β -mediated apoptosis was reduced in cells expressing HCV core as shown by caspase3 activation (Fig. 5C) or loss of mitochondrial membrane potential, which represents another early marker of apoptosis (Fig. 5D).

We then determined EMT process in Huh7 cell lines expressing this core protein. Immunofluorescence studies showed that αSMA was highly polymerized after TGF-β treatment associated with a strong decrease of E-cadherin from the cell membranes (Fig. 5E). αSMA polymerization was increased in core expressing cells. Interestingly, in the presence of core protein, αSMA fibers appeared even in the absence of exogenously added TGF-β. The expression of αSMA was accompanied with anchorage independent growth, which was observed in the absence of exogenously added TGF-β in HCV core protein expressing cells (data not shown).

All together, these data indicate that the effects of HCV core proteins on TGF-β responses observed in primary hepatocytes were reproduced in a human hepatoma cell line that could thus constitute an useful tool to dissect the mechanisms that are involved in the modulation of TGF-β responses.

We also compared protein core expression in our different cellular models and in extracts from liver of HCV/HCC patients. The strongest expression was obtained in human hepatocytes, which is consistent with an efficient lentiviral transduction. HCV core protein expression could be also detected in different liver extracts although at different levels. Interestingly, core expression in these extracts was comparable to the one observed in mouse hepatocytes (Fig. 5F).

### Differential thresholds of Smad3 activation switch TGF-β responses from tumor suppression to tumor promotion

**Figure 6:**
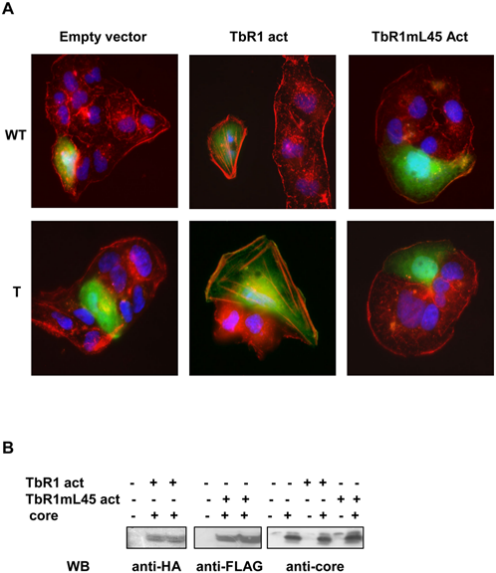
Smad activation is essential to induce TGF-β -mediated EMT. (A) Huh7 cells were co-transfected GFP together with TbR1act or TbR1l45M act plasmids in the presence or absence of HCV core vector. Immunofluorescence analysis was performed 48 h later with an anti αSMA antibody. (B) Expression of TbR1act or TbR1l45M act and HCV core protein were assessed by Western blotting using anti-HA, anti-Flag or anti-core antibodies respectively.

To analyze in more details the contribution of Smad activation in the effects of HCV core on TGF-β responses, we made use of a mutant of the TGF-β receptor I, TβRImL45Act that retains a constitutively active kinase domain but is unable to induce Smad phosphorylation. Huh7 cells were transfected with this mutant or with the wild type activated form of TβRI, together with a plasmid coding for the HCV core and GFP to detect the transfected cells. Immunofluorescence analysis was performed 48 h later. A marked polymerization of αSMA was observed through expression of the constitutively active TβR1 that was similar or even greater when cells also expressed the HCV core protein (Fig. 6A). This effect was completely lost when the cells expressed the TβR1 mutant thus demonstrating the need of activated Smads to initiate EMT.

**Figure 7:**
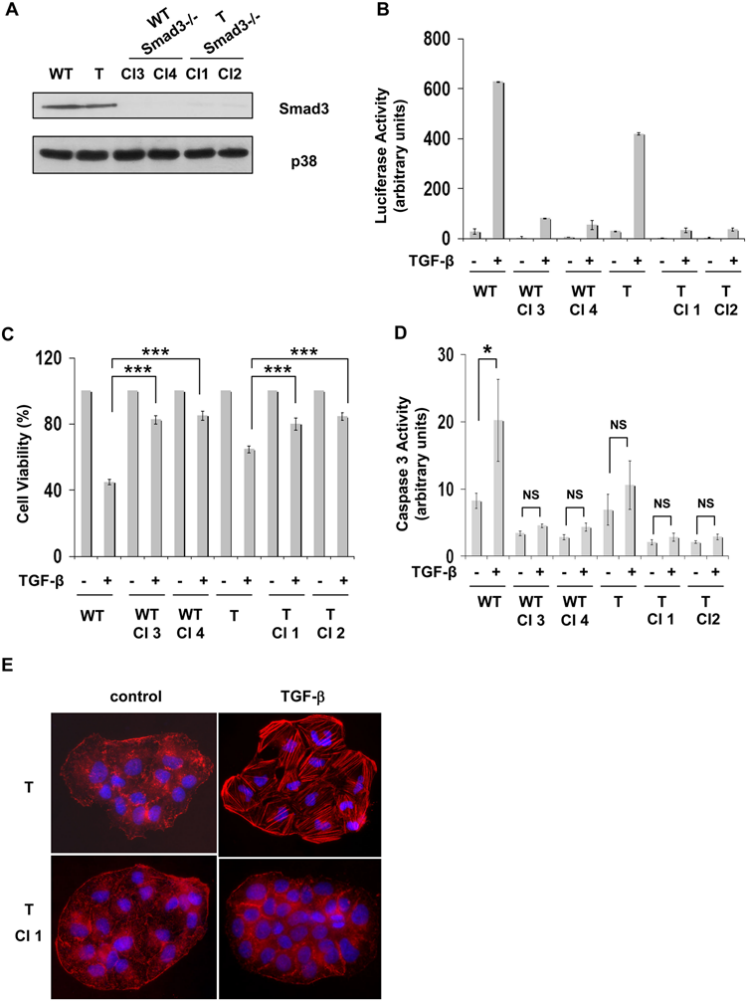
Smad3 depletion prevented TGF-β responses in Huh7 cells expressing or not the HCV core protein. (A) Smad3 expression determined by Western blot analysis in four independent clones selected after stable transfection with pRetroSuper-shRNA-Smad3 plasmid. Anti-p38 antibody was used as control loading. (B) Different clones were transfected with the CAGA-luc reporter plasmid and treated or not with TGF-β (5 ng/ml) for 18 h before determination of luciferase activity. Results were normalized with renilla luciferase and represent the mean of triplicates+/−SD. (C, D) Different clones were treated with TGF-β for 48 h before determination of cell viability (C) or caspase3 activity (D). Results represent the mean+/−SD of triplicates from a representative experiment. * p≤0.05, *** p≤0.0005, NS : not significant. (E) Different clones were treated with TGF-β for 48 h and αSMA polymerization was estimated by immunofluorescence analysis.

To confirm this result, we established different independent Huh7 cell clones, stably expressing or not the HCV core protein, in which the expression of endogenous Smad3 was reduced by stable expression of a short-hairpin RNA (shRNA). As expected, Smad3 depletion prevented TGF-β -induced expression of the CAGA-luc reporter plasmid in the four independent clones tested, two of them expressing the core protein (Fig. 7B). Depletion of Smad3 also blunted the growth inhibitory and apoptotic actions of TGF-β (Fig. 7C and D). Smad3 inactivation also completely blocked TGF-β-induced EMT (Fig. 7E), further supporting the notion that Smad3 plays a crucial role in both tumor suppressor and pro-metastatic effects of TGF-β in carcinogenesis [39].

**Figure 8:**
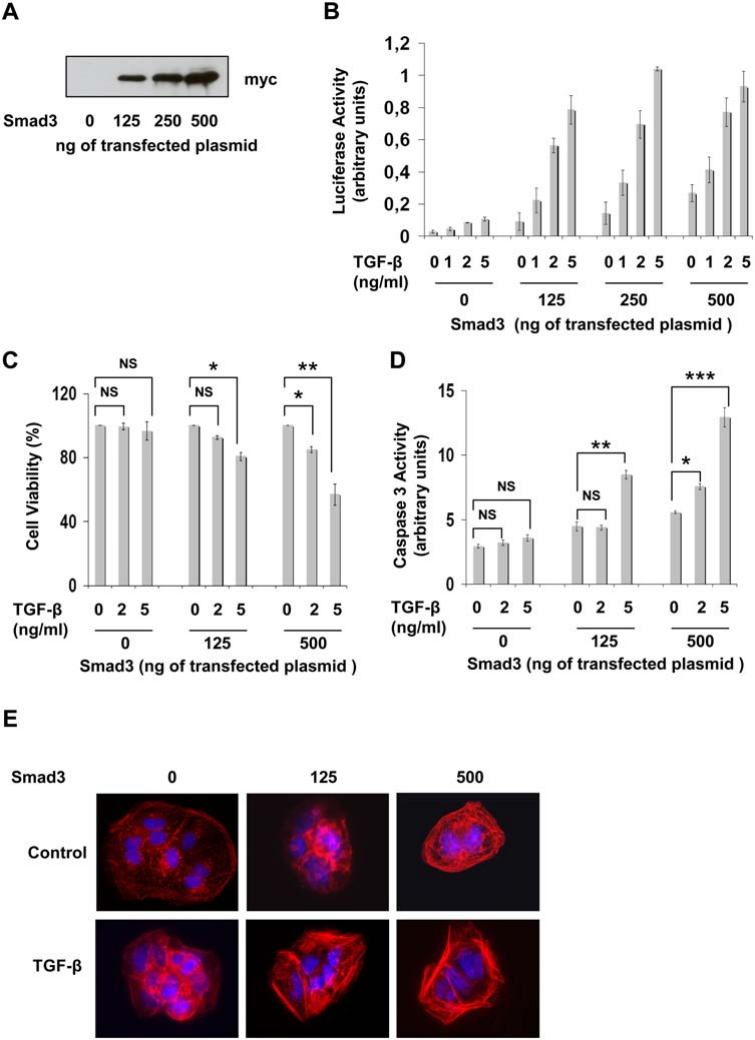
TGF-β responses in Huh7 cells expressing different levels of Smad3. (A) Huh7-shRNA-Smad3 cells (Clone 3) were transfected with increasing amounts of Myc-Smad3 expression vector together with pCMV renilla luciferase. Smad3 protein expression was evaluated 24 h later by Western blot analysis using an anti-Myc antibody and loading was normalized with renilla luciferase expression. (B) Huh7-shRNA-Smad3 cells (Clone 3) were cotransfected with the CAGA-luc reporter plasmid and increasing amounts of Myc-Smad3 vector together with pCMV renilla luciferase. 24 h later, they were treated or not with different doses of TGF-β for 18 h before determination of luciferase activity. Results were normalized with renilla luciferase and represent the mean+/−SD of triplicates from a representative experiment. (C, D) Huh7-shRNA-Smad3 cells (Clone 3) were transfected with increasing amounts of Myc-Smad3 vector together with pGFP plasmid and sorted by FACS 24 h later on the basis of GFP expression. Cells were then cultured for 24 h and treated with different doses of TGF-β for 48 h before determination of cell viability (C) or caspase3 activity (D). * p≤0.05, ** p≤0.005, *** p≤0.0005, NS : not significant. (E) Huh7-shRNA-Smad3 cells (Clone 3) were transfected with increasing amounts of Myc-Smad3 vector together with pGFP plasmid and sorted 24 h later on the basis of GFP expression. αSMA expression was estimated by immunofluorescence analysis after treatment with TGF-β (1 ng/ml) for 48 h.

We next investigated the possibility that different threshold levels of Smad3 contribute to the differential effects of TGF-β on apoptosis or EMT. For this, we reintroduced increasing amounts of Smad3 in Huh7-shRNA-Smad3 clones (Fig 8A) and determined in these cells the levels of TGF-β signaling and anti-tumor or pro-tumor responses. As expected, in cells co-transfected with myc-Smad3 and CAGA-luc reporter plasmids, increasing Smad3 amounts resulted in the amplification of CAGA-luc transactivation after TGF-β treatment (Fig. 8B). Strong Smad3 expression led to consistent luciferase activity in the absence of TGF-β that may be due to constitutive Smad3 activation. To determine TGF-β responses in relation to Smad3 expression, Huh7-shRNA-Smad3 cells were also transfected with different amounts of myc-Smad3 plasmid, together with GFP plasmid and sorted on the basis of GFP expression prior to the addition of TGF-β. Interestingly, when Smad3 was weakly expressed, TGF-β induced apoptosis was only marginal. In these experimental conditions, cell viability was comparable in control and TGF-β treated cells. When higher levels of Smad3 were expressed, decreased cell viability and increased apoptosis could be observed upon TGF-β addition. This is consistent with the notion that a high threshold of Smad3 is necessary to induce TGF-β -mediated anti-tumor responses (Fig. 8C and D).

The GFP positive cells were also analyzed for αSMA expression and polymerization after TGF-β treatment. In contrast with apoptotic data, TGF-β-induced EMT could occur in the context of low Smad3 expression (Fig. 8E). Taken together, these results strongly suggest that the amplitude of Smad3 activation may orientate TGF-β responses towards apoptosis or EMT. These observations could account for the induction of EMT by the HCV core protein despite diminution of TGF-β signaling.

## Discussion

Our study offers relevant observations regarding both the mechanisms of HCV-related carcinogenesis and the impact of TGF-β in human cancer. Indeed, we provide evidence that HCC-derived HCV core proteins alleviate cell growth inhibition and apoptosis mediated by TGF-β indicating a biological significance of the binding of HCV core protein to Smad3. This effect was not restricted to stably transfected cell lines, since it was also observed in primary mouse hepatocytes isolated from transgenic animals expressing the core proteins as well as in primary human hepatocytes infected in vitro with lentiviruses encoding the same variants. Thus HCV core protein has also the potential to negatively impact the cytostatic actions of TGF-β in systems that may better reflect an *in vivo* situation. These data are in agreement with previous results suggesting that Smad3 is a predominant mediator of TGF-β-induced apoptosis. One attractive possibility could be that by interacting with Smad3, HCV core protein set a threshold level of TGF-β signaling that allowed for a modulation of the magnitude of TGF-β cytostatic responses. Consistent with this notion, we observed that overexpression of Smad3 could reverse this effect of HCV core on TGF-β responses in terms of Smad3 signaling, apoptosis and viability (not shown). Furthermore, this effect of HCV core protein on TGF-β cytostatic responses appears to be specific because it was not observed when another apoptotic cytokine such as TRAIL was employed.

Interestingly, in cells expressing HCV core proteins TGF-β was still able to reduce E-cadherin expression and increase αSMA expression and polymerization that are hallmarks of EMT. These alterations were associated with the ability of these cells to exhibit anchorage independent growth. Importantly, we also observed that core protein expression was sufficient to provoke EMT induction in primary hepatocytes. This effect was reverted by addition of a specific inhibitor of TGF-β I receptor thus demonstrating a TGF-β dependent effect of core on EMT development. . These data emphasize a differential effect on TGF-β actions in terms of apoptosis or EMT.

Different levels of HCV core expression have been observed in HCV-derived HCC at the mRNA level or in immunohistochemistry [40]. Using extracts isolated from livers of HCV/HCC patients we could detect core expression at the protein level. Moreover, we have previously shown that core protein extracted from HCV/HCC tumor tissue could bind Smad3 in GST-pull down analyses [27] suggesting that perturbation of TGF-β signaling may also be modulated in vivo. Overall these results are consistent with the hypothesis that this mechanism could operate during the development of HCV-induced HCC. Interestingly, both tumor and cirrhotic tissues-derived mutants demonstrated these biological effects; this likely reflects the preneoplastic nature of most cirrhotic nodules. However, we did observe a more pronounced biological effect of tumor-derived mutant on TGF-β signaling; this might suggest an HCV quasispecies selection in clonally proliferating tumor cells, consistent with our previous analyses [20], [22].

It is commonly accepted that TGF-β signaling pathway plays a tumor suppressor role thought to be associated with growth inhibitory and apoptotic responses and a tumor promoter role thought to reflect the positive effects of TGF-β on tumor cell invasion. Taken together, our data suggest that HCV core, by reducing Smad3 signal strength, renders the cells to become less sensible to tumor suppressive effects of TGF-β although they retain the tumor promoting effects, assuming that Smad3 may regulate different targets in function of its level of activation. This is consistent with the notion that critical signal amplitude may be needed to evoke a biological effect. In addition to Smad pathways, non-smad-dependent signal transduction downstream of TGF-β receptors has been proposed [41], [42]. Among them, the MAP Kinase pathways including ERK, JNK or p38 as well as PI3K/AKT have been shown to be modulated by TGF-β. Since different reports have shown that HCV core protein could also modulate these pathways, alternative mechanisms could also contribute to TGF-β responses leading to tumor promotion.

It has been recently reported that hyperactive Ras mediates a decrease in TGF-β -induced Smad3 phosphorylation in the COOH-terminal and an increase in JNK-induced Smad3 phosphorylation in the linker region, shifting the TGF-β pathway from a tumor suppressive to an invasive capacity in human colorectal as well as hepatic carcinogenesis [43]. Using a different model, our results, relevant for human carcinogenesis, show that reduction of Smad3 activation could account for a tumor promoting role of TGF-β and raise the possibility that core protein may trigger one step of liver carcinogenesis by modulating the balance between TGF-β antitumor or protumor responses.

Although activation and transdifferentiation of hepatic stellate cells are still regarded as key mechanisms of fibrogenesis, recent studies have pointed out that other liver cells, including hepatocytes may contribute to the pool of myofibroblasts in fibrosing liver. Our results showing that TGF-β is able to induce EMT in primary mouse and human hepatocytes add further evidence for this concept. Furthermore, because HCV replicates in hepatocytes, the fact that EMT could develop in HCV core-expressing cells under TGF-β might provide a new notion to explain the fibrotic effect of this virus.

In conclusion, our data ties together TGF-β and HCV which are both known to be keys in the development of fibrosis and HCC, highlight the ability of hepatocytes to develop EMT under TGF-β and emphasize the role of HCV core protein in the dynamic of these effects.

Moreover, one report has suggested EMT as a mechanism of Epstein-Barr virus-related tumor cell dissemination [44]. Adenoviruses [45] or Papilloma viruses proteins have also been reported to interact with Smad3-dependent transcription [46], [47]. Overall, our present results significantly reinforce the hypothesis that TGF-β and EMT are important drivers of virus-induced human cancers.
